# Effects of intrauterine exposures to polychlorinated biphenyls, methylmercury, and lead on birth weight in Japanese male and female newborns

**DOI:** 10.1186/s12199-017-0635-6

**Published:** 2017-04-12

**Authors:** Nozomi Tatsuta, Naoyuki Kurokawa, Kunihiko Nakai, Keita Suzuki, Miyuki Iwai-Shimada, Katsuyuki Murata, Hiroshi Satoh

**Affiliations:** 1 Development and Environmental Medicine, Tohoku University Graduate School of Medicine, Sendai, Japan; 2 Miyagi University of Education, Sendai, Japan; 3 Faculty of Education, Kochi University, Kochi, Japan; 4 National Institute for Environmental Studies, Tsukuba, Japan; 5 Department of Environmental Health Sciences, Akita University Graduate School of Medicine, Akita, Japan; 6 Tohoku University Graduate School of Medicine, Sendai, Japan; 7 2-1 Seiryo-machi, Aoba-ku, Sendai, Miyagi 980-8575 Japan

**Keywords:** Polychlorinated biphenyls, Methylmercury, Lead, Birth weight, Sex difference

## Abstract

**Background:**

The effects of prenatal exposures to polychlorinated biphenyls (PCBs), methylmercury, and lead on birth weight remain disputable. The aim of this study was to investigate whether these chemicals affect birth weight of Japanese newborns, with special emphasis on determining whether these effects differ between males and females.

**Methods:**

The subjects from Tohoku Study of Child Development, which was designed to examine the developmental effects of prenatal exposures to such hazardous chemicals, were 489 mother-newborn pairs with complete data including smoking habit during pregnancy.

**Results:**

The mean birth weight of all newborns was 3083 (range, 2412–4240) g. The median values of biomarkers in cord blood were 46.0 (5th and 95th percentiles, 18.6–113.8) ng/g–lipid for total PCBs, 10.1 (4.3–22.4) ng/g for total mercury (THg), and 1.0 (0.6-1.7) μg/dL for lead. The birth weight was significantly heavier in the 252 male newborns than in the 237 female ones. A negative association between total PCBs and birth weight was observed in both male and female newborns, even after adjusting for possible confounders. However, a negative association of THg with birth weight was found only in the male newborns. There was no significant relationship between lead and birth weight in both groups.

**Conclusion:**

Birth weight appears to be affected by prenatal PCB exposure in Japanese male and female newborns, and the effect of methylmercury exposure on male fetal growth may be stronger than that for females. This implication is that the effects on fetal growth should be assessed in males and females separately.

## Background

Since polychlorinated biphenyls (PCBs) and methylmercury that are environmentally persistent toxicants cross the placenta, they may affect child development. Prenatal lead exposure seems to result in low birth weight [1–6]. However, the effects of PCBs and methylmercury on fetal growth remain controversial [7]: Some studies, performed in developed countries, reported adverse effects of prenatal PCB exposure on birth weight [8–11]; by contrast, one study showed a positive association between them [12]. Others failed to find such a significant association [13–17]. Likewise, Foldspang and Hansen reported that high maternal and offspring methylmercury concentrations were associated with low birth weight [18], but other researchers did not present a significant association between the exposure and outcome [5, 19–21]. Apart from prenatal exposures via fish and seafood intake, the children of heavy and moderate smokers during pregnancy have a higher rate of being small for gestational age (SGA) than those of non-smokers [22]. If the number of cigarettes smoked and passive smoking during pregnancy were not examined in a study [8], absence of the principal cause would confuse a result derived from such toxic substances. Low birth weight is known to be associated with several chronic diseases in adults, including diabetes mellitus and hypertension [23]. For the prevention of adult diseases, therefore, it is crucial to confirm which pollutants affect fetal growth.

In recent years, the exposure levels of environmental pollutants such as PCBs and lead are low in developed countries [24, 25], predicting that the range of exposure (i.e., difference between the minimum and maximum) may not be wide enough to detect a statistically significant dose–response relationship. In this case, use of concurrent exposure model will be desirable for considering the interactive effect of potential substances other than the concerned one, even though the exposure levels were not so high. In fact, essential nutrients such as *n*-3 polyunsaturated fatty acids (PUFAs) masked the effects of methylmercury [26, 27]. Also, two previous studies demonstrated the association between maternal PCB concentrations and low birth weight only in male newborns [28, 29], hypothesizing that male fetuses may be more vulnerable to such toxicants than female ones. Thus, careful selection of study subjects, exposure markers and possible confounders is sought when examining the complicated link between prenatal low-level exposures and fetal growth.

We have been performing a prospective birth cohort study (i.e., Tohoku Study of Child Development, TSCD), focusing both on the potential risks and benefits of fish eating during pregnancy to investigate the effects of toxic chemicals on child development in Japan [30–33]. In this study, we investigated the impacts of PCBs, methylmercury and lead on birth weight in Japanese newborns, with special emphasis on determining whether the effects differed between males and females. At the same time, we reconsidered some confounders that may mislead our result in data analysis.

## Methods

### Study design and subjects

The detailed study design and protocol of TSCD have been already described in our previous paper [34]. The research field was comprised of two areas, an urban area and a coastal area in the northeast region of Japan, but the subjects recruited at obstetrical wards of two hospitals in the urban area (i.e., Sendai City, Miyagi) were employed in this study because the PCB concentrations in cord blood were determined only in that area. To establish an optimal study population, the eligibility criteria included a singleton pregnancy, Japanese as the mother tongue, neonates born at term (36–42 weeks of gestation) with birth weight of more than 2400 g, and no congenital anomalies or diseases. Information about pregnancy, delivery and the characteristics of infants at birth was obtained from medical records. All procedures of this study were approved by the Medical Ethics Committee of the Tohoku University Graduate School of Medicine.

We enrolled 687 women in this study with their written informed consent, in accordance with the Declaration of Helsinki of 1964 as revised in 2000; and, 599 mother-newborn pairs were registered by September 2003 (participation rate, 87.2%). Of them, 489 pairs with complete information on the PCBs, mercury and lead concentrations in cord blood, birth weight, and possible confounders such as gestational age and seafood intake were used in the present study.

### Exposure markers

Umbilical cord blood was collected into a clean bottle immediately after birth. The samples were frozen at -80 ^o^C until analysis. All 209 PCB congeners were analyzed using high-resolution gas chromatography/high-resolution mass spectrometry with the isotope dilution method. The laboratory analytical methods and quality control procedures have been described elsewhere [35]. All sample analyses were performed by IDEA Consultants, Inc. (Tokyo, Japan) and Shimadzu Techno-Research, Inc. (Kyoto, Japan). The quality of PCB analyses in the two laboratories was validated using an external quality assurance program, the German external assurance scheme at IDEA Consultants, Inc., and that of the Fukuoka Institute of Health and Environmental Sciences at Shimadzu Techno-Research, Inc. Accuracy was ensured using a reference serum sample for quality control; the International Union of Pure and Applied Chemistry No.28, 52, 101, 138, 153, 180 of PCB congeners were determined to be 0.310, 0.162, 0.150, 0.220, 0.217, and 0.300 μg/L, as compared to the reference value (tolerance range in parenthesis) of 0.284 (0.181–0.388), 0.162 (0.102–0.222), 0.172 (0.126–0.218), 0.242 (0.174–0.310), 0.217 (0.152–0.281), and 0.307 (0.222–0.392) μg/L, respectively. The PCB data from two laboratories showed no significant difference by paired *t* test (*t* = −2.572, *P* = 0.062) and a high Pearson product-moment correlation coefficient (*r* = 0.869). The calculated limit of detection (LOD) was 0.03 pg/g-wet, which was identified by the signal-to-noise ratio. The amounts of congeners below the LOD were set at zero. We used the lipid basis for the sum concentration of the all measured PCB congeners (ΣPCB), expressed as ng/g-lipid [36].

Total mercury (THg) and lead concentrations in whole cord blood were analyzed by IDEA Consultants, Inc. (Tokyo, Japan), using cold vapor atomic absorption spectrometry for THg and inductively coupled plasma mass spectrometry for lead [30]. Maternal hair samples were also obtained from the back of the head near the occipital protuberance 2 days after delivery, and the THg levels in 3-cm hair close to the scalp were measured by cold vapor atomic absorption spectrophotometry [30]. The data quality for THg and lead concentrations was validated using external quality assurance programs [37].

### Possible confounders

Information about pregnancy, delivery and characteristics of newborns such as maternal height and body weight before pregnancy, gestational age (weeks), delivery type (spontaneous/Caesarean section or vacuum extraction), birth weight and parity (first child or not), was extracted from medical records. The body mass index (BMI, kg/m^2^) before pregnancy was calculated from the height and body weight. We obtained information about demographics and smoking and drinking habits during pregnancy (presence/absence) from a questionnaire 4 days after delivery. At the same time, maternal intake of fish/seafood during pregnancy also was assessed using a food frequency questionnaire that was administered by trained interviewers.

### Data analysis

Sex differences in basal characteristics and exposure levels were analyzed by Student *t* test, Mann-Whitney *U* test or Fisher exact test. The ΣPCB, THg, and lead concentrations in cord blood and fish/seafood intake were logarithmically transformed because of the skewed distribution. Although the ΣPCB and 2,2’,4,4’,5,5’-hexachlorobiphenyl (PCB-153) of major PCB congener measured by two laboratories seemed to be comparable as mentioned above, they were used after the adjustment for the laboratory by using multiple regression analysis. Pearson product-moment correlation coefficients were used to test the relationships between either birth weight or body weight gain during pregnancy and the relating indicators including exposure biomarkers. Multiple regression analysis was employed to adjust for gestational age, parity, BMI before pregnancy, smoking/drinking habits during pregnancy, and fish/seafood intake, whereas parity showed a strong relation to PCBs, i.e., the first child had higher-level PCBs than the other one [35, 38]. In addition, the impact of collinearity among ΣPCB, THg, and fish/seafood intake was measured by the variance inflation factor (VIF): The VIF was calculated for each predictor by doing a linear regression of that predictor on all the other predictors, i.e., VIF = 1/(1 - *R* ^2^), where *R* is the multiple correlation coefficient; and, a predictor with VIF > 10 is considered as an indicative of serious collinearity [39]. All analyses, with two-sided *p* values, were performed using SPSS Ver. 23.0 (SPSS Japan, Tokyo) and the significance level was set at 5%.

## Results

The mean birth weight of 489 newborns was 3083 (range, 2412–4240) g, and the gestational age was 39.5 ± 1.3 (SD) weeks. The median values of biomarkers in cord blood were 46.0 (5th and 95th percentiles, 18.6 - 113.8) ng/g-lipid for ΣPCB, 10.1 (4.3–22.4) ng/g for THg, and 1.0 (0.6–1.7) μg/dL for lead. The median ΣPCB concentration was 0.122 (0.050–0.332) ng/g-wet and the correlation coefficient (*r*) between the wet-base and lipid-base was 0.892 (*p* < 0.001). The median THg concentration in maternal hair was 2.0 (0.9–4.4) μg/g and the *r* between those in hair and cord blood was 0.841 (*p* < 0.001). Since birth weight differed significantly between 252 male and 237 female newborns as shown in Table 1, the following analyses were carried out for the male and female newborns separately.

**Table 1:** Basal characteristics and exposure levels in 489 mother-baby pairs

	Male newborns (*n* = 252)	Female newborns (*n* = 237)	*p* value^a^
	Mean ± SD or median and the 5–95 percentiles (or number and %) in parenthesis	Mean ± SD or median and the 5–95 percentiles (or number and %) in parenthesis	
Maternal characteristics			
Maternal age (years)	31.5 ± 4.3	31.3 ± 4.4	0.497
Body weight before pregnancy (kg)	53.1 ± 7.4	52.6 ± 8.4	0.511
Body mass index before pregnancy (kg/m^2^)	21.0 ± 2.4	20.9 ± 3.3	0.906
Body weight gain during pregnancy (kg)	9.6 ± 3.7	9.8 ± 3.6	0.584
Fish/seafood intake during pregnancy (g/day)	51.6 (13.1–109.2)	53.6 (12.2–131.4)	0.201
Smoking habit during pregnancy (smokers, %)	17 (6.7)	20 (8.4)	0.499
Drinking habit during pregnancy (drinkers, %)	82 (32.5)	73 (30.8)	0.698
Delivery type (spontaneous, %)	176 (69.8)	178 (75.1)	0.225
Newborn characteristics			
Gestational age (weeks)	39.5 ± 1.3	39.6 ± 1.2	0.516
Birth weight (g)	3126 ± 353	3036 ± 314	0.003
Parity (first-born children, %)	133 (52.8)	123 (51.9)	0.857
Biomarkers in cord blood			
Total PCBs (ng/g-lipid)^b^	49.4 (16.3–118.6)	44.6 (19.6–107.5)	0.170
PCB-153 (ng/g-lipid)^b^	10.6 (3.2–27.9)	9.7 (3.9–23.4)	0.243
Total mercury (ng/g)	10.2 (4.5–23.8)	9.9 (4.0–21.4)	0.669
Lead (μg/dL)	1.0 (0.6–1.7)	1.0 (0.5–1.7)	0.252

^a^Student *t* test, Mann-Whitney *U* test, and Fisher exact test were used for significant difference test

^b^These data were used following adjusting for the laboratory

Table 2 represents correlations between either birth weight or body weight gain during pregnancy and the related indicators. In the 489 mother-newborn pairs, gestational age, BMI before pregnancy, and body weight gain during pregnancy showed significant correlations with birth weight; similarly, maternal age, gestational age, and BMI had significant correlations with body weight gain during pregnancy. Notably, birth weight and body weight gain were negatively correlated with ΣPCB and PCB-153 in the male and female newborns, and THg was inversely related to birth weight only in the male newborns.

**Table 2:** Correlation coefficients (*r*) between birth weight and gestational weight gain and the relating indicators

	Birth weight		Body weight gain during pregnancy	
	*r*	*p* value	*r*	*p* value
252 Male newborns				
Gestational age	0.413	<0.001	0.145	0.021
Maternal age	−0.022	0.722	−0.278	< 0.001
Body mass index before pregnancy	0.175	0.005	0.059	0.354
Body weight gain during pregnancy	0.200	0.001	-	
Fish/seafood intake	0.045	0.473	0.029	0.651
Total PCBs in cord blood^a,b^	−0.252	< 0.001	−0.334	< 0.001
PCB-153 in cord blood^a,b^	−0.247	< 0.001	−0.325	< 0.001
Total mercury in cord blood^b^	−0.130	0.039	−0.007	0.917
Lead in cord blood^b^	−0.025	0.698	0.037	0.560
237 Female newborns				
Gestational age	0.311	< 0.001	0.116	0.074
Maternal age	0.081	0.212	−0.127	0.050
Body mass index before pregnancy	0.176	0.006	−0.245	< 0.001
Body weight gain during pregnancy	0.251	0.004	-	
Fish/seafood intake	0.048	0.465	−0.010	0.874
Total PCBs in cord blood^a,b^	−0.170	0.009	−0.270	< 0.001
PCB-153 in cord blood^a,b^	−0.179	0.006	−0.264	< 0.001
Total mercury in cord blood^b^	−0.023	0.720	0.039	0.555
Lead in cord blood^b^	−0.030	0.642	0.126	0.053
Total newborns				
Gestational age	0.361	< 0.001	0.133	0.003
Maternal age	0.029	0.519	−0.208	< 0.001
Body mass index before pregnancy	0.171	< 0.001	−0.103	0.022
Body weight gain during pregnancy	0.190	< 0.001	-	
Fish/seafood intake	0.043	0.348	0.009	0.843
Total PCBs in cord blood^a,b^	−0.207	< 0.001	−0.307	< 0.001
PCB-153 in cord blood^a,b^	−0.209	< 0.001	−0.299	< 0.001
Total mercury in cord blood^b^	−0.074	0.100	0.014	0.763
Lead in cord blood^b^	−0.022	0.635	0.078	0.086

^a^These data were used following adjusting for the laboratory

^b^All biomarkers in cord blood and seafood intake were logarithmically transformed

Relations of prenatal biomarkers to birth weight in the mother-newborn pairs are listed in Table 3. In this table, although parity was significantly associated with birth weight in the total newborns, it was not so in the male or female newborns. In adding two interactive variables of (sex) × (ΣPCB) and (sex) × (THg) in independent variables of the total newborns, the two variables were not significantly related to birth weight (*p* = 0.983 and *p* = 0.373, respectively) but the significance of other variables presented in Table 3 remained unchanged.

**Table 3:** Relations of prenatal biomarkers and confounders to birth weight: results of multiple regression analysis^a^

	252 Male newborns		237 Female newborns		Total newborns	
	Standardized regression coefficient, *β*	*p* value	Standardized regression coefficient, *β*	*p* value	Standardized regression coefficient, *β*	*p* value
Total PCBs in cord blood	−0.171	0.004	−0.166	0.009	−0.161	< 0.001
Total mercury in cord blood	−0.120	0.042	−0.042	0.499	−0.078	0.061
Lead in cord blood	0.023	0.692	−0.039	0.513	−0.011	0.784
Possible confounders						
Gestational age	0.397	< 0.001	0.373	< 0.001	0.383	< 0.001
Parity	0.075	0.201	0.102	0.116	0.093	0.030
Body mass index before pregnancy	0.143	0.012	0.227	< 0.001	0.180	< 0.001
Smoking during pregnancy	−0.066	0.237	0.080	0.185	0.006	0.889
Drinking during pregnancy	0.001	0.986	0.019	0.756	0.010	0.812
Fish/seafood intake	0.080	0.168	0.067	0.288	0.069	0.099
Child sex	-	-	-	-	0.159	< 0.001
Contribution rate, *R* ^2^	0.229	< 0.001	0.204	< 0.001	0.239	< 0.001

^a^Total PCBs was used following adjusting for the laboratory, and all biomarkers in cord blood and fish/seafood intake were logarithmically transformed

*R* indicates the multiple correlation coefficient

As shown in Table 3, lower birth weight was significantly associated with ΣPCB in the male and female newborns, but also with THg only in the male ones. On the other hand, there was no significant association between cord-blood lead and birth weight for either sex. Even when adding body weight gain during pregnancy to independent variables of the multiple regression models, the significance of ΣPCB and THg was almost the same as in Table 3. Concerning the collinearity among exposure markers of 489 newborns, the correlation coefficients were 0.171 (*p* < 0.001) for ΣPCB and THg, 0.069 (*p* = 0.128) for ΣPCB and fish/seafood intake, and 0.239 (*p* < 0.001) for THg and fish/seafood intake; and, the VIFs for ΣPCB, THg, and fish/seafood intake were 1.045, 1.097, and 1.078, respectively.

## Discussion

Given the concurrent exposure model of environmental pollutants, the principal findings of our study were that birth weight was associated with ΣPCB concentrations in the male and female newborns, and also with THg concentrations in the male ones. However, the significant relation to lead was not seen, probably not only because of low lead levels but also the extremely narrow range of lead exposure, as compared to the values reported previously [2–6]; whereas, Taylor and coworkers could not find any evidence suggesting a dose–response relationship either in 4190 births (median of maternal blood lead, 3.40 μg/dL; range, 0.20 - 19.14 μg/dL) [40]. In addition, no evidence of multicollinearity problem among ΣPCB, THg, and fish/seafood intake or of interaction between sex and toxic substances on birth weight was suggested, because all the VIFs were less than 2 in the present study [39]. Thus, a concurrent exposure model appears to be important for the assessment of the effect on fetal growth/SGA, whereas this model did not include interactive variables between these chemicals. Otherwise, the effect of prenatal exposures to a plural pollutant at low levels may be underestimated or ignored.

In the current study, there was a significant negative relationship between cord-blood ΣPCB and birth weight in the 489 newborns, along with in each group of the male and female ones. This is consistent with results of many reports [8–11, 41–43]. Two previous studies observed the negative association only in male newborns [28, 29], and Yamashita and Hayashi confirmed it only in female newborns [44]. However, some reports could not find such a significant association [13–17]. At least, all studies using cord blood as a biological specimen demonstrated the significant association; that is, it may be preferable to use the more direct specimen when compared to maternal blood or milk, inasmuch as maternal and umbilical-cord concentrations in red blood cells do not show a significant correlation for several chemical substances [45]. Taken together, our data support the idea that PCBs can affect fetal growth. The ultimate way to protect fetuses against the harmful effect of PCBs, therefore, is for girls and women not to eat specific fish/marine mammals containing high-level PCBs, like whale blubber, until they have given birth as recommended in dietary advisories of the Faroe Islands [46].

A significant association of cord-blood THg with low birth weight was observed in the male newborns, but not in the female ones, while the impact of methylmercury did not seem to be stronger than that of PCBs judging from *β* values of Table 3. This finding is similar to that reported by Foldspang and Hansen [18], who examined 376 mothers living in Greenland and demonstrated a negative effect of dietary methylmercury on intrauterine growth. By contrast, Obi and coworkers reported that cord-blood mercury was positively associated with birth weight and length [47], which may have been due to the fact that their THg levels were extremely low (median, 4.9 μg/L) and their data were unadjusted for any confounders. However, some studies did not detect any significant relationships between the exposure and birth outcomes [5, 19–21]. If these analyses were done for males and females separately, they might have discovered a significant association. Thus, male birth weight appears to have been affected by prenatal methylmercury exposure at relatively low levels (median, 10.2 ng/g in cord blood). In fact, when the methylmercury pollution in Minamata, Japan, was most severe (i.e., in 1955–1959), decreases in male births were observed in the overall city population, in fishermen, and in maternal Minamata disease patients, and an increase in the proportion of male stillborn fetuses was seen [48]. In other words, male fetuses are suggested to be more susceptible to methylmercury than female counterparts. Sex difference should be kept in mind when assessing reproductive toxicants.

In examining the relationships between environmental pollutants and fetal growth, gestational weight gain is frequently used as a possible confounder [12, 20, 43]. However, body weight gain during pregnancy was significantly correlated with birth weight (Table 2), and it showed close relations to the ΣPCB and PCB-153, as well as gestational age, maternal age, and BMI before pregnancy, which implies that birth weight and gestational weight gain together are outcome variables originating from intrauterine PCB exposure. Verner and coworkers noted that gestational weight gain is an imprecise measure of the increased maternal lipid compartment during pregnancy [43]. A significant association of parity with PCB concentrations is also well-known [35]. In fact, the addition of either gestational weight gain or parity in multiple regression analysis did not change our results when PCB levels existed (Table 3). Accordingly, it is suggested that superficial confounders had better to be excluded from explanatory variables of birth size to avoid misleading or overadjustment.

There may have been some limitations in this study. Although *n*-3 PUFAs are essential for normal brain development, we could not determine the levels because of the limited amount of cord blood available. Instead of *n*-3 PUFAs, we used fish/seafood intake in the data analysis, whereas it is thought to indicate the exposure levels of both beneficial nutrients and toxic substances such as methylmercury and dioxin-like PCBs [27, 49]. Other persistent organic pollutants such as dioxin-like PCBs, polychlorinated dibenzo-p-dioxines, and polychlorinated dibenzofurans, were not analyzed in this study but a significant correlation between dioxin-like PCBs and ΣPCB (*r* = 0.91) was observed in 49 samples of our participants [35]. Likewise, smoking habit during pregnancy was employed as a substitute for the number of cigarettes smoked. There is also a paper describing that birth weight may decrease at high intake levels of marine fats [50], but neither fish/seafood intake nor smoking habit was significantly related to birth weight in the multiple regression analysis as shown in Table 3. As mentioned above, the ΣPCB concentrations in cord blood were determined at two different laboratories, but these values were used following adjustment for the institute. Thus, our data appear not to have been heavily influenced by confounders or measurement bias.

## Conclusion

Birth weight in both male and female newborns appears to be affected by maternal PCB exposure. Intrauterine methylmercury at low levels may also affect male birth weight. Concerning the effect on fetal growth, further studies are necessary to clarify the toxic mechanism of such chemicals, and also to scrutinize sex difference in the outcomes attributable to environmental pollutants other than PCBs and methylmercury.

## Abbreviations

BMI: Body mass index
LOD: Limit of detection
*n*-3 PUFAs: Polyunsaturated fatty acids
PCBs: Polychlorinated biphenyls
SGA: Small for gestational age
THg: Total mercury
TSCD: Tohoku Study of Child Development
VIF: Variance inflation factor
